# Reviving Duran’s Approach to Pericardial Valve Reconstruction in the Pulmonary Position Within the Right Ventricle-to-Pulmonary Artery Conduit: A Compelling Case Report

**DOI:** 10.21470/1678-9741-2024-0123

**Published:** 2025-04-15

**Authors:** Anupam Das, Alok Kumar Sharma, Anirudh Mathur

**Affiliations:** 1 Department of Cardiothoracic and Vascular Surgery, All India Institute of Medical Sciences, Jodhpur, Rajasthan, India

**Keywords:** Pulmonary Atresia, Pulmonary Artery, Congenital Heart Defects, Polyethylene Terephthalates, Adolescent

## Abstract

Various techniques of conduit repair have been employed during valve reconstruction. While Ozaki conduits have streamlined the procedure, their limited availability poses concerns. This case report presents 14-year-old patient with pulmonary atresia and an anomalous left anterior descending artery arising from the right sinus. A right ventricle-to-pulmonary artery conduit was created using Dacron® graft and a trileaflet valve employing Duran's technique of pericardial valve reconstruction, elucidating surgical methodology. In developing countries, the implementation of Duran's technique presents noteworthy advantage allowing for utilization of autologous tissue, addressing challenges associated with PTFE conduits. Unlike PTFE conduits, the results of Duran’s technique at the pulmonary position needs to be followed up in a large number of cases.

## INTRODUCTION

**Table:** 

Abbreviations, Acronyms & Symbols	
BSA	= Body surface area
CT	= Computed tomography
LAD	= Left anterior descending
PTFE	= Polytetrafluoroethylene
RVOT	= Right ventricular outflow tract

The absence of homografts and bovine jugular vein conduits poses a significant challenge for surgeons treating pulmonary atresia. Various techniques, including polytetrafluoroethylene (PTFE) conduits with hand-sewn monocuspid or bicuspid valves, have been used to establish continuity between the right ventricular infundibulum and the branch pulmonary artery confluence. While effective in the short term, these methods require complex calculations during valve leaflet reconstruction. Ozaki conduits have partly simplified the procedure, but their limited availability and the complexity of suturing remain concerns. We report a case of a 14-year-old patient with pulmonary atresia and an anomalous left anterior descending (LAD) artery. With limited availability of expanded PTFE membrane (0.1 mm thickness) and extensive calculations involved in designing them on the table, Duran’s moulds provided an alternative in this report using patient’s autologous tissue for valve reconstruction. A right ventricle-to-pulmonary artery conduit was created using a Dacron® graft, incorporating a trileaflet valve employing Duran's technique of pericardial valve reconstruction, offering a viable alternative to conventional conduits.

## CASE PRESENTATION

A 14-year-old boy presented with easy fatigability and shortness of breath since childhood. On initial evaluation, he was diagnosed to have pulmonary stenosis with atretic pulmonary valve with a tight annulus on echocardiography. Computed tomography (CT) angiography revealed atretic pulmonary annulus with an anomalous LAD artery arising from right sinus having a pre-pulmonary course, thus a surgical plan for conduit repair was proposed.

### Surgical Technique

**Fig. 1:**
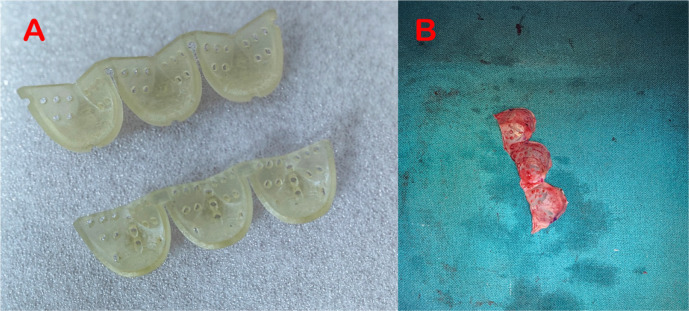
Duran's moulds for pericardial valve cusps tailored for a 21 mm annulus (A); trimmed and secured pericardial valve cusps within Duran's moulds (B).

After sternotomy, a rectangular patch of autologous pericardium was harvested with a length of 1 cm, which was more than three times the expected pulmonary annulus as per body surface area (BSA) of the boy, and a width of at least one diameter and freed from fat and redundant tissue^[1]^. As per the BSA of the boy, a 20 mm Dacron® straight graft was chosen. The pericardium was then placed in a plastic container which had three consecutive bulges of the appropriate size (21 mm Duran’s mould) (Figure 1A)^[1]^ corresponding to the expected pulmonary annulus diameter. The pericardial mesothelial surface was placed in contact with the plastic bulges and held in position with a plastic perforated sheath of the same dimensions as a negative of the bulges^[1]^. The entire arrangement was then fixed with 0.625% glutaraldehyde for 10 minutes at room temperature. The pericardium was then removed and rinsed in 0.9% isotonic saline for three times for 10 minutes and then trimmed down as per the established dimensions (Figure 1B). The lowest point of each commissure was marked.

**Fig. 2:**
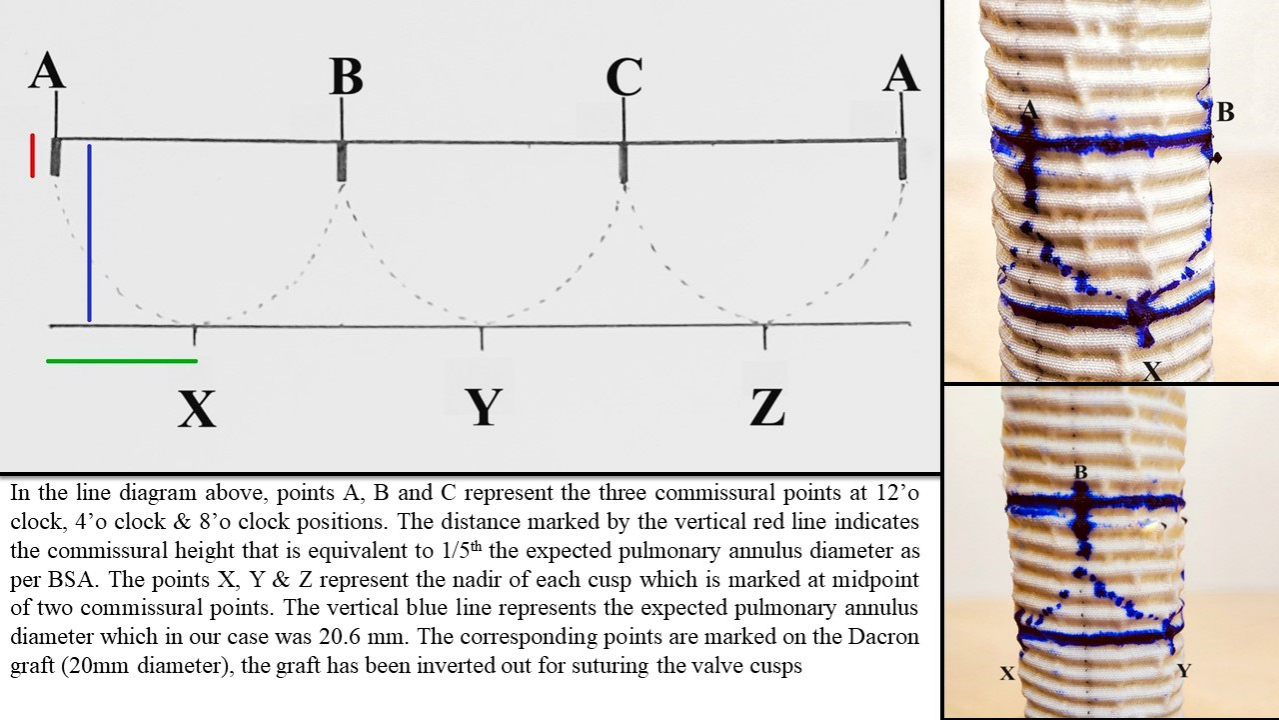
Detailed diagram indicating measurements on the graft and marking points for suturing. BSA=body surface area.

The Dacron® graft was everted inside out. Three equidistant commissural points were strategically marked at 12 o'clock, 4 o'clock, and 8 o'clock. A transverse line was meticulously drawn, linking these three points (points A, B, and C in Figure 2). Another transverse line, situated 2 cm (expected diameter of the pulmonary annulus)^[1]^ below the initial line, was then drawn. Subsequently, the nadir of each cusp was identified on the lower line, precisely at the midpoint between two consecutive commissural points (points X, Y, and Z in Figure 2). Calculating the commissural height as 1/5th of the expected annular diameter^[1]^, corresponding marks were made on the graft. These designated points correlated with the lowest points of each commissure on the pericardial cusps. Three 5-0, 13 mm double-arm polypropylene sutures were utilized to affix the nadir of each cusp to the designated points on the graft. Additionally, three more sutures were employed to secure the highest point of each commissure, aligning them with the corresponding points on the graft (A, B, C). We found that the circumference of the graft taken for conduit repair (as per BSA, using the diameter value which corresponds to Z score 0) was equivalent to the total length of the pericardium used for the valve cusps after final trimming.

**Fig. 3:**
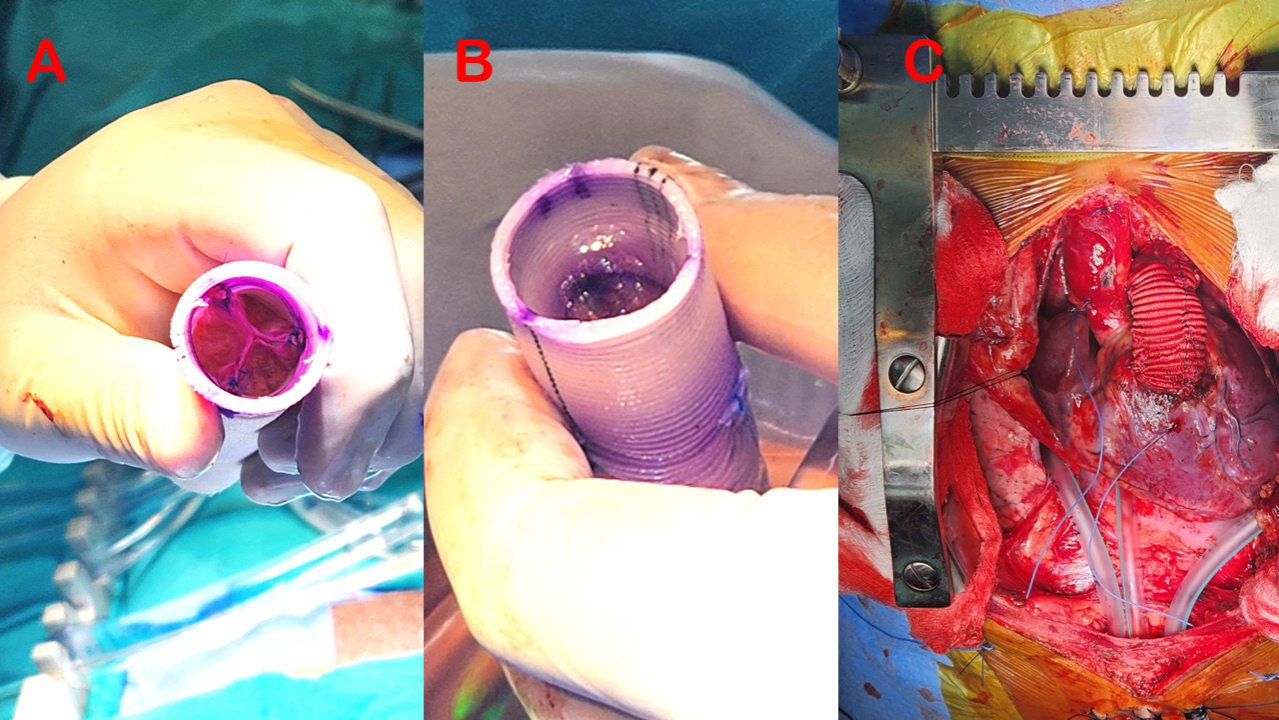
Tricuspid pericardial pulmonary valve within the Dacron® graft (A); saline test to assess valve competency (B); final implantation of the right ventricle-to-pulmonary artery conduit (C).

Suturing was performed in a continuous fashion taking partial thickness bites within the graft to prevent needle hole bleeding. The graft with the affixed valve was then carefully inverted, and the valve cusps were aligned in the centre to rule out any difference in their heights and any redundancy (Figure 3A). Each commissural pillar was additionally strengthened using a 6-0 9 mm double-arm polypropylene suture incorporating 1-2 mm of the free margin of each cusp and passed exactly at the same level of the commissure’s highest point from inside to out and tied using a small Dacron® pledget which took care of any redundancy of the free margins, if any.

**Fig. 4:**
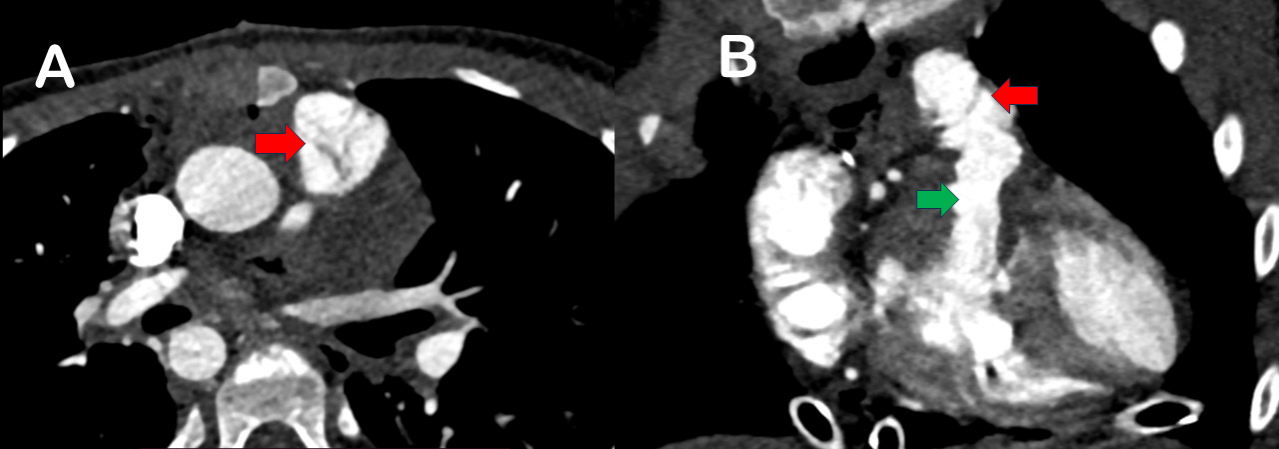
Computed tomography images (A and B) depicting pericardial valve cusps positioning in pulmonary locations (red arrows); wide right ventricular outflow tract (green arrow).

After establishing cardiopulmonary bypass using aorto-bicaval cannulation, diastolic arrest was achieved using DelNido cardioplegia, and right ventriculotomy was performed just proximal to the anomalous LAD artery for a distance of 2 cm, limited by a large conal branch crossing anteriorly on the right ventricular wall. Infundibular resection was performed, the hypoplastic pulmonary artery was divided at the bifurcation, and the atretic annulus was closed in two layers using 4-0 17 mm polypropylene suture. The distal anastomosis of conduit was performed using 5-0 13 mm polypropylene, the proximal conduit was bevelled, and proximal anastomosis was accomplished using 5-10 17 mm polypropylene suture (Figure 3B). After weaning off cardiopulmonary bypass, the postoperative transoesophageal echocardiography revealed a peak gradient across the valve of 18 mmHg with no regurgitation. The boy was discharged on postoperative day five, and after six months, the pericardial valve is shown to function well with no regurgitation and with an acceptable peak gradient of 16 mmHg. CT performed at one-year follow-up showed alignment of tricuspid pulmonary valve and wide right ventricular outflow tract (RVOT), as depicted in Figure 4.

## DISCUSSION

The case presented here underscores the challenges associated with managing pulmonary atresia, especially in the context of limited conduit options. The unavailability of homografts and bovine jugular vein conduits necessitates innovative solutions, and in this case, we revisited Duran's approach to pericardial valve reconstruction for a compelling alternative. Duran's technique of pericardial valve reconstruction offers a practical solution, utilizing readily available moulds to craft a trileaflet valve^[1]^. While alternative methods involving PTFE conduits with hand-sewn valves have shown short-term success, they often involve complex calculations or assumptions during the reconstruction of pulmonary valve leaflets^[2]^. The adoption of Ozaki conduits has mitigated some complexity, but their limited availability remains a concern in terms of measurement of annulus and creating leaflets from the template to create three equal-size leaflets to optimize coaptation height and distribute equal tension throughout the valve^[3]^. However, Brown et al.^[4]^ described excellent long-term results of the use of PTFE as a monocusp valve in RVOT; it diminished initial and midterm pulmonary insufficiency without significant stenosis as alternative for RVOT conduit.

Duran’s moulds were designed based on previously published work on the aortic valve^[5-8]^; their use in the pulmonary position is a novel technique, the long-term efficacy of which needs to be seen in a larger cohort. 3D printing technology can be used to replicate these moulds, providing for their wide availability without significant increase in the surgical costs. The meticulous steps taken, including glutaraldehyde fixation and careful trimming, ensured the creation of a valve that met the specific anatomical requirements. It offers a reproducible and effective method for creating a competent pulmonary valve in challenging anatomical scenarios. The use of autologous pericardium and reusable moulds make this technique feasible where alternative conduits may be limited^[9]^. The presence of a Dacron® graft may also provide for a scaffolding for transcatheter therapies should the pericardial valve develops regurgitation or stenosis in the future. While the presented case demonstrates the feasibility and success of Duran's approach, it is essential to acknowledge potential limitations. Larger studies and long-term follow-up are needed to validate the durability and performance of this technique across a diverse patient population. Duran’s technique at the aortic position provided an actuarial survival of 84.53% ± 12.29% at 60 months, freedom from failure of the aortic reconstruction of 83.83% ± 8.59%, and freedom from any event of 72.59% ± 12.79% at five-year follow-up^[10]^. Unlike for aortic valve repair, wherein the mechanical stress is considerable, at the pulmonary position, this technique may be expected to give good results in the long term. However, the potential for calcification of these pericardial leaflets can’t be ruled out.

## CONCLUSION

In conclusion, the revival of Duran's approach to pericardial valve reconstruction in the pulmonary position within the right ventricle-to-pulmonary artery conduit may offer a reproducible solution in the absence of conventional conduits with the replication of the moulds using modern 3D printing technology which needs to be explored.
